# Therapeutic Strategies Targeting the Kidney–Liver–Immune–Heart Network: Circadian and Mechanosensory Pathways in CKD-Associated Cardiac Injury

**DOI:** 10.3390/ijms27083436

**Published:** 2026-04-11

**Authors:** Yuya Yoshida, Kohei Fukuoka, Tomohito Tanihara, Kengo Hamamura, Akito Tsuruta, Satoru Koyanagi, Shigehiro Ohdo, Naoya Matsunaga

**Affiliations:** 1 Department of Clinical Pharmacokinetics, Faculty of Pharmaceutical Sciences, Kyushu University, 3-1-1 Maidashi Higashi-ku, Fukuoka 812-8582, Japanohdo@phar.kyushu-u.ac.jp (S.O.); 2 Department of Pharmaceutics, Faculty of Pharmaceutical Sciences, Kyushu University, 3-1-1 Maidashi Higashi-ku, Fukuoka 812-8582, Japan

**Keywords:** chronic kidney disease, heart failure, cardio–renal syndrome, circadian rhythm, macrophage, DBP, vitamin A, retinoids, mechanotransduction, GPR68, PIEZO1, TRPV4, TRPC6

## Abstract

The present review discusses vitamin A/retinoid metabolism as a cross-organ axis in which hepatic clock-dependent retinoid handling may affect immune clock gene expression through the stimulation of retinoic acid 6–Janus kinase 2–signal transducer and activator of transcription 5 signaling, potentially promoting pro-inflammatory monocyte states. We further highlight mechanosensory signaling as a second convergent layer that integrates hemodynamic forces with tissue microenvironmental cues. Among these pathways, G protein-coupled receptor 68, a proton- and flow-sensitive G protein-coupled receptor, is discussed as a representative druggable node linking mechanical and inflammatory signaling in chronic kidney disease-associated cardiac injury. Finally, we outline potential therapeutic directions, including (i) circadian alignment/chronopharmacology, (ii) modulation of retinoid metabolism and signaling, and (iii) targeted inhibition of primary immune and mechanosensory effectors.

## 1. Introduction

Heart failure (HF) remains a leading cause of morbidity and mortality worldwide. Chronic kidney disease (CKD) is one of the most prevalent and prognostically significant comorbidities of HF. Reduced estimated glomerular filtration rate and worsening renal function are consistently associated with increased risks of HF hospitalization and death across cohorts and meta-analyses, highlighting kidney dysfunction as a central determinant of HF outcomes [1]. Current guidelines emphasize the integrated management of HF and its comorbidities, including CKD. However, therapeutic decision-making in CKD is often limited by altered pharmacokinetics, hyperkalemia risk, and susceptibility to drug-induced kidney injury [2,3].

Conceptually, cardiorenal syndrome (CRS) describes a spectrum of bidirectional interactions in which acute or chronic dysfunction of the heart or kidney induces dysfunction in the other organ. In addition to hemodynamic coupling, CRS encompasses neurohormonal activation, inflammation, oxidative stress, and metabolic dysregulation [4,5]. Emerging evidence further suggests that CRS may represent a broader multi-organ network disease extending to the liver and immune system—both of which may modulate systemic metabolism and inflammatory tone.

CKD is associated with significant alterations in cardiovascular structure and function, including left ventricular hypertrophy, interstitial fibrosis, impaired diastolic function, and ultimately HF. CKD-associated cardiac injury is not only a hemodynamic problem but also an immune–metabolic disorder characterized by chronic, sterile inflammation and maladaptive tissue remodeling. These processes are increasingly recognized to be modulated in part by circadian clocks, and their disruption is increasingly recognized as a systemic risk factor across organ systems, including kidney and eye diseases [6]. In addition, mechanosensory signaling may contribute to these inflammatory processes by translating physical forces and tissue microenvironmental cues into inflammatory gene programs.

Chronopharmacology and chronotherapy provide a translational framework for leveraging rhythmic variations in drug targets, pharmacokinetics, and toxicity to enhance efficacy and safety [7,8]. Several standard treatments based on chronopharmacology have demonstrated sufficient clinical efficacy and are widely used in practice; these include HMG-CoA reductase inhibitors for dyslipidemia and prednisolone for rheumatoid arthritis [9,10]. In cardiovascular medicine, large-scale trials testing bedtime dosing of antihypertensives have yielded divergent results, as exemplified by the Hygia Chronotherapy and Treatment In Morning versus Evening (TIME) trials, highlighting both potential opportunities and ongoing controversies [11,12]. In addition to blood pressure control, time-of-day effects have been observed in diverse immunopharmacological contexts, including diurnal regulation of immune checkpoints and antibiotic-induced kidney injury in preclinical models [13,14]. Collectively, these observations provide a rationale for considering chronobiological and network-based therapeutic strategies in CKD-associated cardiac injury.

In the present review, we distinguish relatively well-established principles in circadian and retinoid biology from emerging cross-organ mechanisms that remain to be more broadly validated.

## 2. An Emerging Kidney–Liver–Immune–Heart Network Framework for CKD-Associated Cardiac Injury

Cardio–renal interactions have been extensively investigated within the CRS framework, which emphasizes bidirectional hemodynamic and neurohormonal coupling, as well as inflammatory and metabolic pathways [3,4,5]. However, accumulating evidence suggests that CKD-associated cardiac impairment may arise, at least in part, from a broader network of inter-organ communication rather than from kidney dysfunction alone. In this emerging framework, renal injury may alter circulating factors, including cytokines, metabolites, and hormones. These alterations, in turn, could reshape hepatic metabolic programs and immune cell states, influencing monocyte/macrophage recruitment and activation in the heart. Experimental CKD models, including prior research from our group, have demonstrated that myeloid circadian machinery is remodeled and that monocyte/macrophage-specific disruption of the core clock component aryl hydrocarbon receptor nuclear translocator-like 1 (brain and muscle ARNT-like 1 [BMAL1]) can attenuate cardiac inflammation and fibrosis, despite persistent systemic risk factors [15,16]. These findings support the biological plausibility of a kidney–liver–immune–heart network, although broader validation across models and human disease settings remains necessary.

### 2.1. Monocyte/Macrophage Clocks as Potential Disease Modifiers

Immune cells exhibit intrinsic circadian clocks that regulate inflammatory responsiveness, pathogen defense, and tissue trafficking [17,18]. In addition to bone marrow release, the spleen can serve as a reservoir of monocytes. These monocytes are mobilized to inflamed tissues, forming a systemic circuit for myeloid trafficking [19]. CKD is characterized by chronic low-grade inflammation and complex immune dysfunction, affecting both innate and adaptive compartments. Uremic retention solutes and microbiota-derived metabolites can reprogram monocytes and other immune cells, contributing to cardiovascular risk [20]. In this context, peripheral clocks within myeloid cells act as transcriptional regulators of inflammatory tone, metabolic wiring, and tissue trafficking. In CKD, monocytes can acquire pro-inflammatory programs that accelerate cardiac remodeling. Mechanistic studies have associated CKD with altered clock gene expression in monocytes and macrophages and increased cardiac infiltration of inflammatory myeloid cells [15]. These findings suggest the therapeutic potential of the myeloid clock; Modulating clock components may suppress the inflammatory output without compromising essential host defense functions.

### 2.2. Hepatic Clock Output and Systemic Metabolic Signaling

Beyond the kidney, CKD exerts systemic effects on hepatic function, including altered expression and activity of drug-metabolizing enzymes and transporters in the liver and intestine. Uremic toxins and inflammatory cytokines can downregulate cytochrome P450 enzymes and transport systems, thereby modifying non-renal drug clearance and endogenous metabolite handling [21,22,23]. The liver is a central hub for rhythmic metabolism. CKD can disrupt hepatic clock output by altering endocrine and inflammatory signals. Notably, CKD-associated activation of transforming growth factor beta signaling can suppress hepatic D-box-binding protein (DBP), which is a clock-controlled transcription factor, thereby downregulating the expression of retinoid-metabolizing enzymes and aggravating renal dysfunction in experimental models [24,25]. As hepatic metabolites and transport proteins circulate systemically, CKD-driven hepatic clock disruption may influence immune and cardiovascular function through circulating metabolites and transport pathways, thereby providing a plausible mechanism for multi-organ disease amplification.

## 3. Circadian Coordination of Renal, Immune, and Cardiovascular Physiology

This section first summarizes relatively well-established circadian principles in renal and immune physiology and highlights downstream inflammatory pathways that remain less well validated in CKD.

### 3.1. Renal Tubular Transport, Peripheral Kidney Clocks, and Inflammation-Related Pathways in CKD

The kidney exhibits robust circadian regulation of electrolyte transport, acid-base balance, and urine concentration. For example, the expression of the Na^+^/H^+^ exchanger 3 is directly regulated by the CLOCK:BMAL1 machinery and oscillates in renal tubular segments [26]. Through these clock-controlled transport pathways, the renal circadian system governs sodium handling, blood pressure rhythms, and systemic metabolic homeostasis [27,28,29]. Accordingly, disruption of kidney clocks is implicated in abnormalities of tubular transport and broader pathophysiological processes driving CKD progression. Beyond intrinsic tubular functions, circadian disruption in CKD is increasingly recognized as a systemic phenomenon. Clinical observations in patients undergoing hemodialysis have revealed disturbed peripheral circadian rhythms, supporting the notion that CKD is associated with altered temporal organization at the whole-body level rather than in the kidney alone [30]. Experimental and review studies have highlighted links between renal clock dysfunction, hypertension, fibrosis, and metabolic dysregulation [27,28,29]. Together, these findings support the general concept that circadian disturbance contributes to CKD pathophysiology; however, the specific inflammatory mediators and cell type-specific pathways downstream of renal clock disruption remain incompletely understood.

G0/G1 switch 2 (G0S2) represents a candidate pathway relevant to circadian-driven renal inflammation. We have previously revealed that G0S2 is associated with renal inflammation in experimental CKD [24]. Beyond the kidney, G0S2 broadly serves as a regulator of lipid metabolism through its interaction with adipose triglyceride lipase (ATGL), with previous studies establishing its role in controlling lipolysis, hepatic energy balance, and metabolic adaptation [31,32]. In addition, modulation of the ATGL-associated adipose inflammatory axis influences cardiac remodeling, suggesting that G0S2-related metabolic pathways may extend beyond adipose tissue to cardio-metabolic inflammation [33]. While these findings provide biological plausibility for considering G0S2 as a metabolism-linked inflammatory modifier, current evidence does not establish G0S2 as a universally established central regulator of CKD inflammation. In this review, we highlight G0S2 as a candidate node within a broader network linking circadian disturbance, metabolic rewiring, and inflammatory responses, rather than as the definitive mechanism explaining CKD-associated renal or cardiorenal injury. Similarly, although clock-related signaling may intersect with inflammatory pathways, such as NF-kappaB, the causal hierarchy and generalizability of these interactions remain to be clarified across different CKD models and human disease settings.

Thus, a balanced interpretation is that circadian dysfunction is increasingly supported as a contributor to CKD pathophysiology, whereas specific downstream pathways, including G0S2-linked inflammatory or metabolic signaling, remain emerging mechanisms that warrant further independent validation. Future studies using human biospecimens, longitudinal rhythm phenotyping, and cell type-resolved analyses are required to determine which clock-controlled pathways drive primary disease and which represent secondary adaptive responses to chronic kidney injury. Critical unresolved questions include whether CLOCK/G0S2/NF-kappaB-linked signaling acts as a causal driver, a context-dependent modifier, or a secondary consequence of broader inflammatory remodeling in CKD.

### 3.2. Neural–Immune Interfaces and Rhythmic Leukocyte Trafficking

In CKD, neural–immune trafficking pathways may be particularly relevant because chronic uremic inflammation and altered neurohumoral signaling can shift the timing and magnitude of leukocyte mobilization. Notably, rhythmic C–X–C motif chemokine ligand 12 expression and C–X–C motif chemokine receptor 4 signaling may be reinforced by sympathetic nervous system inputs and local endothelial clocks; together, these components regulate leukocyte trafficking between the bone marrow and circulation [34,35,36]. In addition, circadian programs operate at mucosal immune interfaces in the gut, including gut-associated lymphoid tissues, such as Peyer’s patches [37]. Beyond local mucosal rhythms, intrinsic clocks within inflammatory monocytes and macrophages regulate inflammatory gene programs and the magnitude of innate immune responses [33,38,39,40]. This indicates that circadian dysregulation in CKD may amplify systemic inflammation through both trafficking- and cell-intrinsic mechanisms, providing a plausible basis for enhanced myeloid mobilization and cardiac infiltration under chronic sterile inflammatory conditions.

### 3.3. Clock Nuclear Receptors as Immune Switches

**Figure 1:**
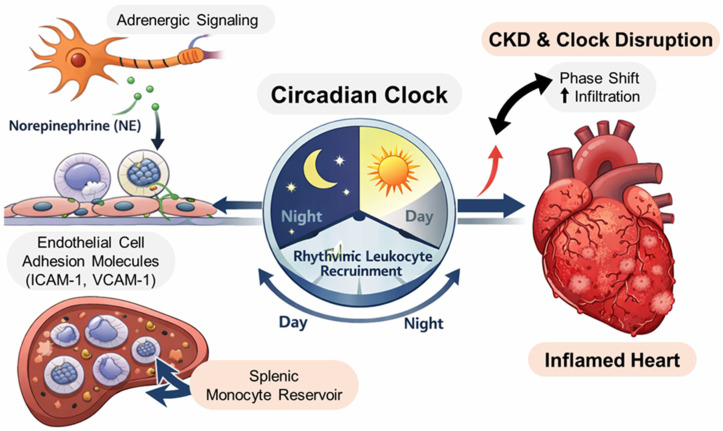
Circadian and neural regulation of leukocyte trafficking relevant to chronic kidney disease (CKD)-associated cardiac inflammation. Adrenergic nerves and endothelial adhesion programs regulate rhythmic leukocyte recruitment, whereas splenic monocyte reservoirs provide rapidly mobilizable inflammatory cells. CKD-induced clock disruption may shift the amplitude/phase of these rhythms and increase cardiac myeloid infiltration.

Clock nuclear receptors act as integrative switches that couple circadian timing with immune effector programs. In macrophages and related myeloid populations, reverse-erythroblastosis (viral) oncogene B (REV-ERB)alpha/beta and retinoic acid receptor–related orphan receptor family members connect core clock oscillations to inflammatory transcriptional outputs, including enhancer-level control of cytokine programs and basal inflammatory tone [41,42,43]. Notably, these nuclear receptors also serve as entry points for systemic signals, such as inflammatory cytokine-nuclear factor kappa B activation, glucocorticoids, metabolic cues, and neurohumoral pathways. These signals can reset or re-weight immune clocks and reshape inflammatory competence [44,45,46]. Moreover, in this broader entrainment network, prostaglandin-mediated signals and neurotransmitter receptor pathways modulate clock gene expression in cellular and organismal contexts [47,48]. In CKD, these regulatory nodes may be particularly relevant, as persistent inflammatory signaling, metabolic disturbance, and endocrine imbalance can re-weight immune clock outputs, shifting responses toward maladaptive inflammatory competence rather than normal rhythmic immune adaptation (Figure 1). This conceptual link becomes especially important when considering retinoid- and metabolism-associated signals discussed below, which may intersect with clock nuclear receptor function in disease-specific contexts.

## 4. Vitamin A/Retinoid Signaling Across the Kidney–Liver–Immune–Heart Network

This section summarizes relatively well-supported aspects of retinoid transport and metabolism and discusses emerging hypotheses regarding cross-organ immune programming in CKD.

### 4.1. Retinol Transport and Stimulated by Retinoic Acid 6–Janus Kinase 2–Signal Transducer and Activator of Transcription 5 (STRA6–JAK2–STAT5) Signaling

**Figure 2:**
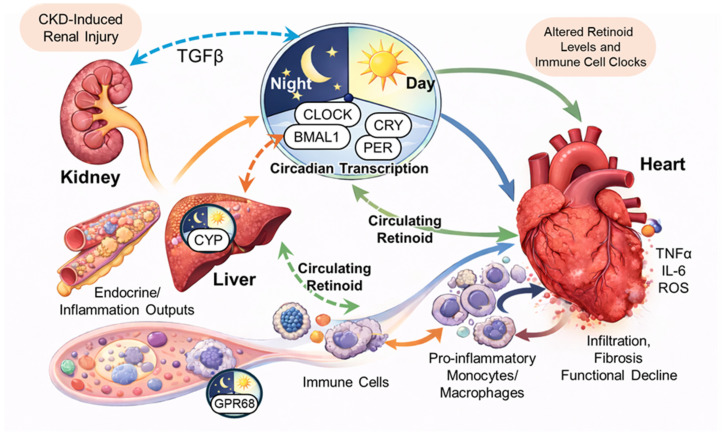
Schematic overview of the kidney–liver–immune–heart network in chronic kidney disease. Renal injury may disrupt endocrine or inflammatory outputs that remodel hepatic circadian transcription and retinoid metabolism, potentially altering circulating retinoids and immune cell clocks. Pro-inflammatory monocytes or macrophages may subsequently be mobilized and infiltrate the heart, where they facilitate inflammation, fibrosis, and functional decline.

Vitamin A circulates primarily as retinol bound to retinol-binding protein 4 (RBP4) that also functions as a metabolic signal implicated in insulin resistance and systemic inflammation [49]. Cellular uptake can occur via the membrane receptor STRA6, originally identified as a receptor for RBP–retinol complexes [50] and later structurally resolved to reveal the primary elements of the retinol-uptake machinery [51]. STRA6 couples retinol transport to JAK2–STAT signaling. STRA6-mediated retinol signaling can regulate gene expression and systemic metabolic responses through the JAK2/STAT5 pathway [52,53]. In CKD, this signaling pathway provides a plausible mechanism by which altered retinol availability may influence immune cell state and potentially contribute to circadian reprogramming (Figure 2).

### 4.2. CKD-Associated Alterations in Vitamin a Homeostasis: Evidence from Other Groups

CKD disrupts vitamin A (retinol) homeostasis, including elevations in circulating RBP4 and altered tissue retinoid storage/metabolism in renal dysfunction [54,55,56,57]. In experimental work from our group, hepatic clock output disruption involving DBP was associated with broad alterations in hepatic metabolic gene expression, suggesting a possible mechanism for systemic retinoid imbalance in CKD [25]. Beyond these observations, inflammatory signals reshape hepatic retinoid metabolism at the level of retinoic acid synthesis and catabolism. For example, lipopolysaccharide can antagonize retinoic acid-induced expression of the retinoic acid–hydroxylating enzymes cytochrome P450 family 26 subfamily A member 1 (CYP26A1) and CYP26B1 [58], and cytochrome P450-dependent retinoic acid clearance is widely recognized as a primary control point for retinoid signaling intensity [59]. Notably, retinoid nuclear receptors, including retinoid X receptor that forms heterodimers with numerous metabolic nuclear receptors, have been discussed as components that intersect with the molecular clockwork in the liver; this provides a conceptual framework for how circadian disruption can translate into altered retinoid signaling and downstream cardio–renal outcomes [60]. Although these observations support a conceptual link between circadian disruption and retinoid dysregulation, direct validation of this axis in human CKD and in immune cell-specific contexts remains limited.

### 4.3. Retinoid Metabolism in HF and Cardiac Remodeling

In addition to CKD, cardiac retinoid signaling is implicated in HF. Proteomic and metabolomic analyses of failing human hearts and experimental models have demonstrated a reduction in resident cardiac all-trans retinoic acid levels, indicating that local retinoid deficiency may develop during HF progression [61]. Complementary studies have indicated that retinoid signaling can affect cardiomyocyte maturation and survival after injury [62,63]. Notably, pharmacological modulation of retinoid receptors (retinoid X receptor agonists) can attenuate fibroblast activation and post-infarction remodeling in experimental settings [64]. Combined with reports of CKD-associated systemic retinoid disturbances, these observations suggest that both excess and deficiency of retinoid signaling—depending on tissue compartment and disease stage—may modulate cardiac inflammation and fibrosis in the broader cardiorenal context. This duality highlights the need for precision approaches, including stratification by circulating retinoids, hepatic metabolism, and cardiac retinoid signature, before pursuing retinoid-targeted therapy. However, direct evidence linking cardiac retinoid imbalance to CKD-specific inflammatory remodeling in humans remains limited.

### 4.4. Retinoid Signaling in Immune Programming: Current Evidence and Unresolved Questions

Retinoid biology provides a plausible mechanistic link between hepatic metabolism, immune cell state, and cardiovascular remodeling. Independent studies have established that vitamin A/retinoid homeostasis is altered in CKD and that retinoid signaling regulates broad transcriptional and metabolic programs through nuclear receptors and retinoid-responsive pathways [54,56,58,59,65]. Furthermore, retinol transport through RBP4/STRA6 and downstream JAK/STAT signaling can influence cellular gene expression and systemic metabolic responses [50,51,52,53]. More recently, retinol administration was demonstrated to promote the development of HF with preserved ejection fraction after CKD in mice through JAK/STAT signaling. These findings support the concept that retinol-linked signaling can contribute to cardiorenal disease progression beyond our own experimental framework [66]. In addition, other studies support the broader concept of clock-dependent inter-organ communication relevant to kidney disease. For example, loss of the liver circadian clock can alter the phase of intrarenal renin-angiotensin system component expression, indicating that hepatic rhythmicity can influence renal molecular programs even when the precise mediators remain unresolved [67]. Although this previous study did not specifically address retinoid metabolism, it independently validates the general principle that liver-derived circadian signals can shape kidney physiology and therefore strengthens the rationale for considering cross-organ temporal coordination in CKD.

However, the model proposed in this review—that CKD-associated disruption of hepatic clock output reshapes systemic retinoid handling, which reprograms immune circadian machinery and contributes to cardiac inflammation—remains an emerging integrative hypothesis rather than a conclusively established pathway. While research from our group and others supports individual components of this framework, direct causal validation across the full kidney–liver–immune–heart axis remains limited. In particular, evidence directly linking altered retinoid metabolism to monocyte or macrophage circadian reprogramming in CKD is sparse, and the relative contribution of immune versus non-immune retinoid actions remains unresolved. This distinction is important because retinoid signaling is highly context-dependent: depending on the tissue compartment, disease stage, ligand availability, and downstream receptor usage, retinoid pathways can exert anti-inflammatory, differentiation-promoting, or maladaptive effects [53,58,59,61,62,63,64]. Similarly, while the study by Liu et al. supports a disease-promoting effect of retinol in a specific CKD-associated HFpEF model, it does not establish a uniform pathogenic role of retinoids across all CKD-associated cardiorenal settings. Rather, these findings suggest that quantitative and tissue-specific imbalances in retinoid signaling may influence disease phenotypes bidirectionally depending on context.

Therefore, the retinoid-immune-heart framework should be interpreted as a testable disease model integrating hepatic metabolic dysregulation with immune and cardiac consequences, not as the sole or definitive mechanism underlying CKD-associated cardiac injury. Future work should prioritize independent validation in human cohorts and complementary animal systems, focusing on circulating retinoid profiles, liver-derived metabolic signatures, immune cell transcriptional states, and tissue-specific retinoid activity. Such studies will be essential to distinguish well-supported retinoid biology from more speculative cross-organ immune programming mechanisms and determine whether retinoid-targeted interventions can be translated into precision therapeutic strategies.

## 5. Mechanosensing and Tissue Microenvironmental Cues as Convergent Drivers

In CKD-associated cardiac injury, mechanosensory pathways are more appropriately regarded as candidate amplifiers that may integrate altered hemodynamics, tissue stress, and inflammatory signaling, rather than as uniformly established primary disease drivers (Table 1).

### 5.1. G Protein-Coupled Receptor 68 (GPR68) as a Druggable Node Within a Mechanosensor Network

GPR68 (also known as OGR1) is a proton-sensing GPCR that responds to extracellular acidification [68]. Recently, it has been identified as a flow-sensitive receptor essential for vascular physiology, supporting the concept that GPCRs can act as mechanosensors [69]. In experimental CKD, increased GPR68 expression in monocyte-derived macrophages infiltrating the heart is associated with enhanced cardiac inflammation and fibrosis [15]. Pharmacological inhibition of GPR68 (using homoharringtonine in preclinical studies) attenuates CKD-associated cardiac impairment [70]. In addition to homoharringtonine, other groups have developed chemical tools to modulate GPR68 activity. Medicinal chemistry efforts around the positive allosteric modulator ogerin have yielded ogerin-based analogs and clarified structure–activity relationships for GPR68 modulation [71]. In parallel, the ogremorphin class of small-molecule inhibitors (ogremorphin-8345) suppresses GPR68-dependent endothelial dysfunction and inflammatory responses in preclinical models of lung injury [72]. These studies support the pharmacological targeting of GPR68 while highlighting that it represents only one druggable node within a broader mechanosensor network; its contribution to CKD-associated cardiac injury may vary by cell type, disease stage, and local inflammatory or hemodynamic context.

### 5.2. Mechanosensitive Ion Channels: Piezo-Type Mechanosensitive Ion Channel Component 1 (PIEZO1), Transient Receptor Potential Vanilloid 4 (TRPV4), and Transient Receptor Potential Canonical 6 (TRPC6)

Mechanically activated ion channels translate forces, such as stretch, pressure, and shear stress, into Ca^2+^-dependent signaling that can reshape inflammatory and fibrotic gene programs. PIEZO1 and PIEZO2 were identified as core components of mechanically activated cation channels [73]. PIEZO1 is essential for sensing cyclical forces in innate immune cells and can drive inflammatory responses in vivo [74]. In the kidney, TRPV4 functions as a mechanical transducer in flow-sensitive segments of the collecting duct system and participates in flow-dependent signaling [75]. TRPC6, a Ca^2+^-permeable channel expressed in podocytes and other cells, is associated with proteinuric kidney disease and is an emerging therapeutic target; early clinical development of TRPC6 inhibitors (BI 764198) is underway [76,77,78]. Moreover, mechanosensitive channels contribute to cardiac hypertrophy and fibroblast activation, indicating that targeted modulation may provide kidney- and heart-directed benefits [79]. In CKD, these channels may be particularly relevant, as chronic alterations in tubular flow, vascular stiffness, pressure overload, and the uremic inflammatory milieu can reshape mechanical inputs across both renal and cardiovascular tissues.

### 5.3. Endothelial and Cytoskeletal Mechanotransduction Modules

Endothelial cells integrate shear stress through multi-component mechanosensory complexes. A junctional complex, including platelet endothelial cell adhesion molecule-1, vascular endothelial-cadherin, and vascular endothelial growth factor receptor 2 mediate endothelial responses to fluid shear stress [80]. Downstream, cytoskeletal tension and matrix stiffness regulate transcriptional co-activators Yes-associated protein/transcriptional co-activator with PDZ-binding motif (YAP/TAZ) that function as central effectors of mechanotransduction [81]. Other mechanotransduction modules relevant to the kidney and vasculature include integrin-based focal adhesions [82] and primary cilia–polycystin complexes that respond to fluid shear [83]. In the cardio–renal setting, these pathways may couple CKD-associated vascular dysfunction, endothelial stress, and altered hemodynamics with immune cell recruitment, barrier dysfunction, and tissue remodeling. Notably, this offers a plausible link between mechanical stress and chronic sterile inflammation.

### 5.4. Mechanosensitive GPCR Signaling Beyond GPR68

Several GPCRs can respond to mechanical stress, either directly through conformational alterations or indirectly via membrane tension and cytoskeletal coupling. A classic example is the angiotensin II type 1 receptor that can be activated by mechanical stress independently of angiotensin II, contributing to hypertrophic signaling [84]. Similarly, apelin receptor functions as a dual receptor that integrates ligand- and stretch-dependent signaling during cardiac hypertrophy [56]. These findings highlight the broader concept that GPCRs can act as force sensors and may provide additional drug targets for CKD-associated cardiac remodeling. In CKD-associated cardiorenal remodeling, GPCR-dependent force sensing may be particularly relevant under hypertensive conditions, vascular stiffening, and chronic volume or pressure stress; however, direct evidence in CKD-specific inflammatory settings remains limited.

**Table 1:** Selected mechanosensors and mechanotransduction modules implicated in kidney–immune–heart pathology, and representative therapeutic approaches.

Node	Primary Stimulus	Primary Cell Types	Proposed Roles in Chronic Kidney Disease–Heart Axis	Therapeutic Angle	Primary Refs.
G protein-coupled receptor 68	Acidic pH; flow/shear	Monocytes/macrophages; endothelium	Candidate amplifier of inflammatory activation and remodeling	Small-molecule inhibition (preclinical)	[68,69,70,71,72]
Piezo-type mechanosensitive ion channel component 1	Cyclic pressure; stretch	Innate immune cells; endothelium	Force-sensing pathway potentially linked to inflammatory programs	Context-dependent modulation (risk of broad effects)	[74]
Transient receptor potential vanilloid 4 (TRPV4)	Flow; osmotic/mechanical stress	Renal collecting duct; vascular cells	Mechanotransduction in renal tubules and vascular function	Selective TRPV4 inhibitors/antagonists (preclinical/clinical)	[75]
Transient receptor potential canonical 6 (TRPC6)	Receptor-operated Ca^2+^ entry; mechanical inputs	Podocytes; vascular/cardiac cells	Proteinuria and glomerular injury; potential cardio–renal benefit	TRPC6 inhibitors (clinical development)	[76,78]
Platelet endothelial cell adhesion molecule-1/vascular endothelial-cadherin/vascular endothelial growth factor receptor 2 complex	Shear stress	Endothelium	Mechanosensory complex implicated in adhesion and leukocyte trafficking	Target downstream signaling (safer than receptor blockade)	[80]
Yes-associated protein/transcriptional co-activator with PDZ-binding motif (YAP/TAZ)	Matrix stiffness; cytoskeletal tension	Fibroblasts; endothelium; immune cells	Candidate transcriptional effector of fibrosis and mechano-inflammation	Inhibit YAP/TAZ-driven transcription (careful safety)	[81]
Angiotensin II type 1 receptor	Mechanical stress	Cardiomyocytes; vascular cells	Hypertrophic signaling independent of AngII	Inverse agonists/angiotensin II receptor blockers; mechanosensitive bias	[84]

## 6. Therapeutic Strategies Targeting the Kidney–Liver–Immune–Heart Network

Therapeutic opportunities within the kidney–liver–immune–heart network vary markedly in translational maturity. Some approaches, such as chronopharmacological optimization of existing cardiovascular therapies, have been tested in clinical settings, whereas others, including retinoid-directed interventions, myeloid clock reprogramming, and mechanosensor-targeted strategies, remain largely preclinical or hypothesis-driven in CKD-associated cardiac injury. Distinguishing these levels of evidence is important to avoid overinterpretation and to clarify which concepts are ready for clinical evaluation and those that currently serve as mechanistic frameworks for future therapeutic development.

### 6.1. Clinically Explored Chronopharmacological Approaches

Within this broader network framework, therapeutic timing is the most clinically advanced strategy, as it can be applied to already approved interventions without requiring new drug development [8,9]. In cardiovascular medicine, pragmatic clinical trials have assessed morning-versus-evening administration of antihypertensive agents. However, the results have not been fully concordant, as exemplified by the TIME and Hygia studies [10,11]. These data indicate that chronopharmacological optimization is clinically testable and potentially relevant to patients with CKD-associated cardiovascular disease, although a universal bedtime-dosing strategy cannot be currently recommended.

In CKD, implementing chronotherapy will likely require individualized consideration of circadian phase, blood pressure pattern, renal function, and treatment adherence. Moreover, preclinical studies have revealed time-of-day differences in drug sensitivity and toxicity in kidney-relevant pharmacological contexts, including vancomycin-induced kidney injury and circadian regulation of drug metabolism [13,57]. Thus, chronopharmacology represents a clinically informed and partially clinically tested strategy that still requires CKD-specific biomarker-guided validation before routine precision implementation.

Clinicians often find it challenging to establish circadian disruption as a concrete clinical issue and incorporate chronobiological principles into routine practice. Circadian influences are often difficult to measure directly at the bedside, and their relevance may be obscured by interindividual variability, comorbidities, and practical treatment schedule constraints. However, time-conscious treatment strategies have already emerged and gained broad clinical acceptance; these include the timing of glucocorticoid administration and the scheduling of antihypertensive therapy in selected settings. Thus, while further accumulation of rigorous clinical evidence remains essential, progress also depends on training clinicians with sufficient expertise to interpret circadian evidence and educate multidisciplinary clinical teams about when time-of-day effects are likely to be clinically meaningful. Such efforts may help bridge the gap between chronobiological concepts and real-world clinical decision-making.

### 6.2. Retinoid-Directed Strategies: Mechanistically Supported but Not Yet Clinically Established

Given that CKD disrupts hepatic retinoid metabolism and circulating retinoid homeostasis [65], retinoid-focused strategies represent a biologically plausible therapeutic direction. Potential approaches include dietary optimization, modulation of hepatic retinoid-metabolizing enzymes, or selective targeting of retinoid receptors in immune cells or cardiac tissue. A recent study linking retinol-JAK/STAT signaling to CKD-associated HFpEF supports the disease relevance of retinoid-linked pathways in the cardiorenal setting [66]. However, retinoid-directed therapy in this context remains preclinical and conceptually exploratory. Current evidence does not yet define which patients would benefit from retinoid supplementation, restriction, or receptor-selective modulation, nor does it identify validated biomarkers for treatment stratification. In addition, the effects of retinoid signaling are highly context-dependent, varying according to tissue compartment, disease stage, and downstream receptor usage [53,54,59,61,62,63,64,85]. Therefore, retinoid-targeted intervention should presently be considered a mechanistically supported but unvalidated therapeutic concept rather than a clinically validated strategy for CKD-associated cardiac injury.

### 6.3. Myeloid Clock-Targeted Interventions: Preclinical Opportunities

Interventions that selectively reprogram monocytes or macrophages by modulating circadian regulators or downstream inflammatory transcriptional programs may suppress CKD-associated cardiac fibrosis while preserving essential host defense functions. Candidate approaches include manipulation of REV-ERB-related pathways and time-of-day-optimized immune interventions [14,41,42]. However, these strategies remain preclinical; there are no clinically established therapies that specifically target myeloid circadian machinery in CKD-associated cardiac disease. Moreover, important translational questions, including cell type specificity, delivery methods, safety, and durability of immune reprogramming, remain unresolved. Therefore, myeloid clock-targeted therapy represents a promising experimental direction and not a near-term clinical option; safe translation will require cell-selective delivery and preservation of essential host defense in a population that is susceptible to infection and treatment-related complications.

### 6.4. Mechanosensor-Targeted Therapies: Emerging Preclinical Strategies

Targeting mechanosensory pathways offers additional opportunities to suppress inflammation and fibrosis driven by abnormal hemodynamic forces and tissue stress. Candidate approaches include inhibition of GPR68 as a proton- and flow-sensing amplifier of macrophage activation [69,70,71,72], modulation of mechanosensitive ion channels (e.g., PIEZO1, TRPV4, and TRPC6) [71,72,73,74,75,76], and interference with downstream mechanotransduction modules (e.g., YAP/TAZ) [81]. Despite strong mechanistic rationale, most of these approaches remain at the preclinical stage in the context of CKD-associated cardiac injury. Among them, TRPC6 inhibition is the most translationally advanced, with early clinical development in proteinuric kidney disease [77,78]. Nevertheless, its relevance to the broader kidney–liver–immune–heart network and cardiac inflammatory remodeling remains to be established. In contrast, GPR68 inhibition and YAP/TAZ-directed approaches remain supported mainly by preclinical evidence only. Therefore, mechanosensor-targeted therapies should be considered emerging experimental strategies; even where clinical development exists in kidney disease, their efficacy and safety in CKD-associated cardiac inflammatory remodeling remain unproven.

### 6.5. Combination Strategies and Precision Patient Stratification

The network model suggests that single-node intervention may prove insufficient, as CKD-associated cardiac remodeling likely reflects heterogeneous combinations of hemodynamic stress, circadian disruption, metabolic imbalance, and immune activation. In principle, combination strategies, such as circadian alignment together with targeted suppression of a mechanosensory or inflammatory effector, may offer synergistic benefit. Nevertheless, such approaches remain hypothetical. Their development requires patient stratification based on clinically actionable biomarkers, including rhythm metrics, circulating retinoid profiles, immune phenotypes, and markers of fibrosis or vascular stress. Thus, precision combination therapy should be viewed as a forward-looking translational framework rather than a validated therapeutic paradigm. Near-term translational steps may include biomarker-led observational studies, feasibility trials of time-conscious optimization of existing therapies, and prospective stratification of patients by rhythm metrics, inflammatory phenotype, and renal-cardiac risk profile.

Overall, among the strategies discussed in the present review, chronopharmacological optimization is the most readily testable in clinical settings, whereas retinoid-, immune clock-, and mechanosensor-directed interventions remain largely preclinical and require substantial validation before clinical translation.

## 7. Conclusions and Future Directions

CKD-associated cardiac injury arises from a kidney–liver–immune–heart network in which circadian and mechanosensory pathways converge to amplify the inflammation and fibrosis. In this review, we distinguish well-established principles in circadian and retinoid biology from emerging cross-organ mechanisms supported in part by studies from our group and therefore requiring broader independent validation. This framework expands therapeutic opportunities beyond classical hemodynamic control and indicates that effective treatment may require (i) restoring or exploiting circadian organization, (ii) correcting cross-organ metabolic axes, such as vitamin A/retinoid handling, and (iii) selectively targeting mechanosensors and downstream mechanotransduction programs in disease-relevant cell types. Future studies should focus on causal validation in human cohorts, developing actionable biomarkers, and rigorously assessing the safety of clock- and mechanosensor-directed therapies in CKD.

This review has certain limitations. First, many of the mechanisms discussed are derived primarily from animal, ex vivo, or otherwise reductionist experimental systems; their conservation across human CKD phenotypes, disease stages, and treatment settings remains uncertain. Second, translating this framework into routine care requires careful attention to practical limitations; bridging the gap between the academic framework presented in this review and the realities of clinical practice will be essential. For example, controlling vitamin A-related factors in patients receiving maintenance hemodialysis may require blood purification modalities that incorporate convective clearance, such as online hemodiafiltration; however, access to these technologies remains regionally uneven in many healthcare settings. Similarly, time-of-day-based therapeutic interventions face practical constraints related to staffing patterns, routine clinical workflows, and the limited flexibility of treatment schedules in real-world care. Third, many molecular pathways discussed in this review are likely influenced by interindividual variation in circadian phase, genetic polymorphisms, and metabolic background, including variability in enzymes involved in vitamin A metabolism. However, these factors are not yet easily captured by simple, clinically deployable diagnostic tools. Addressing these limitations requires advances in therapeutics, biomarker development, and healthcare systems and implementation infrastructure. In this context, drug development targeting mechanosensors themselves may offer one practical advantage by overcoming difficulties associated with direct measurement or manipulation of complex circadian and metabolic states. While these reflect long-term progress, near-term progress will likely rely on prospective human cohort studies, biomarker-guided patient stratification, and feasibility-focused clinical trials that test time-conscious use of existing therapies before more complex pathway-directed interventions are attempted.

Advancing this field will require integrative approaches that link causal biology across organs over time. Key priorities include (i) defining patient-relevant network phenotypes using longitudinal biomarkers (retinoid profiles, immune cell transcriptional states, and rhythm metrics), (ii) delineating cell type-specific mechanisms using spatial and single-cell approaches, and (iii) evaluating combination strategies that target metabolic inputs, immune programming, and mechanotransduction nodes while preserving essential host defense and homeostatic functions. This precision, network-oriented framework may ultimately enable therapies that more effectively suppress cardiac inflammation and fibrosis in CKD and enhance long-term outcomes.

## Data Availability

No new data were created or analyzed in this study. Data sharing is not applicable to this article.

## Abbreviations

The following abbreviations are used in this manuscript:

ARNTL/BMAL1	Aryl hydrocarbon receptor nuclear translocator-like 1/Brain and muscle ARNT-like 1
ATGL	Adipose triglyceride lipase
BMAL1	Brain and muscle ARNT-like 1
CKD	Chronic kidney disease
CRS	Cradiorenal syndrome
DBP	D-box-binding protein
GPCR	G protein-coupled receptor
HF	Hear failure
PIEZO1	Piezo-type mechanosensitive ion channel component 1
RA	Retinoic acid
RBP	Retinol-binding protein
RBP4	Retinol-binding protein 4
STRA6	Stimulated by retinoic acid 6
STRA6–JAK2–STAT5	Retinoic Acid 6–janus kinase 2–signal transducer and activator of transcription 5
TIME	Treatment In Morning versus Evening (TIME)
TRPC6	Transient receptor potential canonical 6
TRPV4	Transient receptor potential vanilloid 4
